# Consequences of *Campylobacter jejuni* and *Campylobacter coli* Colonisation of Piglets on Gut Microbiota and Microbial Metabolites

**DOI:** 10.3390/microorganisms14050945

**Published:** 2026-04-22

**Authors:** Alexandra Rath, Silke Rautenschlein, Janina Rzeznitzeck, Michael Lalk, Karen Methling, Daniela Karasova, Ivan Rychlik, Karl-Heinz Waldmann, Alexandra von Altrock

**Affiliations:** 1 Clinic for Swine and Small Ruminants, Forensic Medicine and Ambulatory Service, University of Veterinary Medicine Hannover, Foundation, 30173 Hannover, Germanyalexandra.von.altrock@tiho-hannover.de (A.v.A.); 2 Clinic for Poultry, University of Veterinary Medicine Hannover, Foundation, 30559 Hannover, Germany; silke.rautenschlein@tiho-hannover.de (S.R.);; 3 Institute of Biochemistry, University of Greifswald, 17489 Greifswald, Germany; lalk@uni-greifswald.de (M.L.); methling@uni-greifswald.de (K.M.); 4 Veterinary Research Institute, 621 00 Brno, Czech Republicrychlik@vri.cz (I.R.)

**Keywords:** pig, *Campylobacter*, experimental infection, intestine

## Abstract

*Campylobacter* (*C.*) *jejuni* and *C. coli* are common zoonotic bacteria in pigs, which typically act as asymptomatic carriers. However, the effects of *Campylobacter* colonisation on the porcine intestinal microbiota and metabolome remain poorly understood. This study investigated microbiome and metabolome alterations associated with co-colonisation by *C. jejuni* and *C. coli* in the different intestinal segments of pigs. Thirty-two weaned piglets were assigned to a control group and a group inoculated with *C. coli* ST5777/CT828 and *C. jejuni* ST122/CT206. Four weeks post inoculation, jejunal and caecal contents were analysed for *Campylobacter* counts, metabolite profiles and microbial composition. All animals remained clinically healthy. Both *Campylobacter* species colonised the jejunum and caecum, with higher *C. coli* counts in the caecum. *Campylobacter*-colonised pigs showed significantly altered metabolite profiles, including reduced cysteine and urea and increased glycine in the jejunum, as well as elevated 3-hydroxybutyrate levels in the caecum. In contrast, short-chain fatty acid concentrations in the caecum were unaffected by infection. Microbiota analysis revealed a significant reduction in caecal alpha diversity, whereas jejunal diversity remained unchanged. Infected pigs exhibited increased relative abundances of Lactobacillaceae and Bifidobacteriaceae and a decreased abundance of Pseudomonadota, including Enterobacteriaceae. In conclusion, *Campylobacter* co-colonisation induces distinct microbiome and metabolome alterations in pigs despite the absence of clinical disease. These findings highlight complex host-microbiota–pathogen interactions that may be relevant for future *Campylobacter* control strategies in pig production.

## 1. Introduction

Since 2005, campylobacteriosis has been reported as the most common foodborne bacterial enteritis disease in the European Union (EU), with 148,181 confirmed cases in 2023 [1]. In the vast majority of cases, diarrhoea occurs after consuming poultry meat contaminated with *Campylobacter* (*C.*) *jejuni* (74%). However, pork is also considered a potential vector, mainly of *C. coli* (10%) [2].

The occurrence of *Campylobacter* in swine populations is considered to reach as high as 100%, with *C. coli* being significantly more dominant than *C. jejuni* [3,4], although co-infections were proven in 28% of 50 investigated pig herds [5]. In an American study, the number of *C. jejuni* findings even outweighed that of *C. coli* in a pig herd [6].

*C. jejuni* and *C. coli* are both zoonotic pathogens with a broad host range. However, the use of multilocus sequence typing (MLST) revealed that certain sequence types (ST) have a broader host repertoire than others due to a good adaption to different conditions in the gut, representing a host-generalist lifestyle [7].

Pathogenicity of *Campylobacter* is determined by motility, chemotactic orientation, adhesion and invasion ability, as well as toxin production [8]. In contrast to the relevance in humans, intestinal colonisation with *Campylobacter* leads to clinical asymptomatic carriage in most mammals and birds, although it was proven that *C. jejuni* is able to increase intestinal permeability and to translocate from the intestine to internal organs in chickens [9,10,11,12] and in turkeys [13]. In weaned pigs, an impact on the chloride transport mechanism of the caecal epithelium was also noted [14]. Translocation of *C. coli* to gut-associated lymphoid tissues, the tonsils, spleen and gall bladder was observed, while neither clinical symptoms nor histopathological changes in the intestinal wall were detectable [15]. In contrast, gnotobiotic as well as colostrum-deprived neonatal pigs develop clinical enteritis after inoculation with *C. jejuni* [16,17,18,19].

Therefore, the question concerning the distinct outcomes of *Campylobacter* colonisation in humans as well as in avian and mammalian species still remains unanswered.

*C. jejuni* and *C. coli* colonise the small as well as the large intestine of pigs, whereby the detection rate of *C. jejuni* in the anterior intestinal segments exceeds that of *C. coli* [14]. The reason for the different distribution of *Campylobacter* spp. may result from their different nutrient requirements, so that colonisation of certain intestinal sections depends on the available substrates [20,21,22]. Further host factors, in particular the composition of the enteric microbiota, appear to influence the outcome of a colonisation [23,24,25,26], whereas metabolome and microbiome cannot be considered separately. Microbiota-derived metabolites can also be used by *Campylobacter* [27]. Since the microbiota composition changes along the intestine with greater microbial diversity in the large intestine compared to the small intestine [25,28,29,30], metabolome profiles also differ in the intestinal sections [27]. For example, the small intestine contains plenty of monosaccharides and some oligosaccharides, while the large intestine mainly comprises complex carbohydrates, like resistant starch and lignin that are indigestible in the small intestine [30,31]. Due to anaerobic bacterial fermentation of those dietary fibres, short-chain fatty acids (SCFAs) represent the primary metabolic products formed in the large intestine [32].

The interaction with the gut microbiota and its metabolites is expected to affect *Campylobacter* colonisation. Although in recent years the characterisation of the microbiome of the pig intestine has been the subject of numerous studies using modern sequencing approaches [33,34], little is known about the influence of *Campylobacter* colonisation on the composition of the microbiome in pigs. Corresponding investigations have already been carried out in poultry and revealed modifications in the caecal microbial richness in infected birds [13,35,36,37,38].

We hypothesised that, due to the *Campylobacter* infection, both the composition and abundance of the microbiota and the metabolome profile will change in the various sections of the porcine intestine.

Therefore, in the present study, we investigated the colonisation pattern of *C. coli* ST5777/CT828 and *C. jejuni*: ST122/CT206 in pigs after inoculation, taking the intestinal environment into account, to gain new insights into the significance of these influences.

Observations of changes in both the microbiota composition and the composition of various metabolites in the intestinal segments as a result of *Campylobacter* colonisation provide information about the requirements of the infectious strains in their environment. Conversely, this may provide a basis for deriving preventive measures against this zoonotic pathogen by specifically influencing the intestinal ecosystem, e.g., through feeding strategies.

Our study provides new insights into the impact of microbiome–metabolome interactions on *Campylobacter* colonisation in the pig gut.

## 2. Materials and Methods

### 2.1. Campylobacter Strains and Preparations for Inoculation

Two *Campylobacter* strains were employed in this study, as previously reported by Rath et al. [39]: *C. coli* ST5777/CT828 and *C. jejuni* ST122/CT206. Both isolates originated from poultry and have been repeatedly associated with human infections (http://pubmlst.org/campylobacter/ accessed on 18 May 2021).

Distinct antibiotic resistance profiles, generated under laboratory conditions, allowed the strains to be differentiated when cultured on agar supplemented with the respective antibiotics. Specifically, *C. coli* ST5777 exhibits resistance to nalidixic acid, whereas *C. jejuni* ST122 is resistant to streptomycin.

For the infection trial, bacterial cultures were prepared by inoculating culture broth (No. 2, CM0067, Oxoid/Thermo Scientific Inc. Waltham, MA, USA) with colonies of the respective *Campylobacter* species 48 h prior to administration. The cultures were incubated at 37.4 °C under microaerophilic conditions (5% O_2_, 10% CO_2_, and 85% N_2_) using Thermo Scientific™ Oxoid™ CampyGen™. A target infectious dose of 10^8^ colony-forming units (CFU) per animal in 10 mL of broth was intended, and the actual dose was subsequently verified by serial dilution (Table 1).

### 2.2. Rearing of the Animals

A total of 32 piglets (16 males and 16 females) of a crossbreed [(Danish Landrace × German Large White) × German Landrace] were included in the study. All animals were maintained and handled in compliance with the German Animal Welfare Act, which aligns with the guidelines of the German Research Council and the European Council Directive 2010/63/EU governing animal experimentation. Prior to inoculation, and subsequently for Group 0, the absence of *Campylobacter* spp. was confirmed through routine rectal swab analyses. After inoculation, stringent hygiene measures were implemented to prevent cross-contamination between experimental units. Detailed information on animal husbandry and housing conditions has been previously provided by Rath et al. [14].

In the eighth week of life, the piglets were anaesthetised using azaperone (2 mg/kg body weight; Stresnil^®^, ElancoTM, Elanco Inc., Greenfield, IN, USA) in combination with ketamine (20 mg/kg body weight; Ketamine^®^, CP-Pharma^®^, CP-Pharma Handelsgesellschaft GmbH, Burgdorf, Germany). Inoculation was then carried out via a gastric tube (CH 12, B. Braun Melsungen AG) following the experimental design outlined in Table 1.

At necropsy, performed four weeks post-inoculation, the animals exhibited a mean body weight of 30.7 ± 6.4 kg.

### 2.3. Collection and Culture of Campylobacter Isolates from Intestinal Contents

Samples were collected from the contents of the caecum and jejunum at predefined anatomical sites, specifically from the distal tip of the caecum and approximately the third metre of the jejunum.

The procedure for quantifying *Campylobacter* in the intestinal contents has been described previously [39]. Briefly, ingesta samples were cultured on *Campylobacter*-selective charcoal cefoperazone deoxycholate agar (CCDA; Thermo Fisher Scientific, Inc., Waltham, MA, USA). Incubation was carried out for 48 h at 37.4 °C under microaerophilic conditions (5% O_2_, 10% CO_2_, and 85% N_2_) using Thermo Scientific™ Oxoid™ CampyGen™. For post-inoculation differentiation of the strains, CCDA plates were supplemented with the respective antibiotics (nalidixic acid or streptomycin).

To determine bacterial counts, serial tenfold dilutions of the samples were prepared in phosphate-buffered saline (PBS). Each dilution was plated in duplicate onto CCDA agar and incubated under the conditions described above. Following incubation, colony numbers were recorded, and bacterial concentrations (cfu/mL) were calculated in accordance with the standard method (ISO 10272-2:2017).

### 2.4. Metabolomic Profiling of Intestinal Contents

#### 2.4.1. Processing and Preparation of Intestinal Samples

For metabolomic investigations, intestinal content from the jejunum and caecum was collected during necropsy. The ingesta samples were immediately frozen at −80 °C and stored until further processing. Before transport to the Institute of Biochemistry at the University of Greifswald (Germany), samples were thawed on ice. From each sample, 300 mg was transferred into a 2 mL SafeSeal tube (SARSTEDT AG & Co. KG; Nümbrecht, Germany, REF. 72.695.400).

Afterwards, 1 mL of distilled water was added, and the mixture was homogenised using a vortex mixer for 30 s. The samples were then centrifuged at 10,000× *g* for 10 min at 0 °C. The resulting supernatant was carefully collected and passed through a sterile filter (Filtropur S, SARSTEDT AG & Co. KG, Nümbrecht, Germany, No. 83.1826.001) into an Eppendorf reaction tube. Finally, the filtrates were stored again at −80 °C until shipment for subsequent analysis.

#### 2.4.2. ^1^H NMR—Proton Nuclear Magnetic Resonance Spectroscopy

SCFAs were detected using 1H-NMR. Spectroscopic 1H-NMR analysis and quantification were performed as previously described by Rath et al. [39]. In brief, 400 µL of each sample was combined with 200 µL of a 0.2 mol/L sodium hydrogen phosphate buffer prepared in 30% D_2_O (Euriso-Top, St-Aubin Cedex, France), containing 1.74 mmol/L 3-trimethylsilyl-(2,2,3,3-D4)-1-propionic acid (TSP; Sigma-Aldrich, St. Louis, MO, USA). The mixture was centrifuged at 13,000 rpm and 4 °C.

The resulting supernatants were transferred into 5 mm NMR tubes (103.5 mm length; Bruker Biospin GmbH, Rheinstetten, Germany) and analysed at 300 K. Measurements were carried out using a Bruker AVANCE NEO 600 spectrometer equipped with a SampleJet autosampler and a 5 mm QCI cryoprobe, operated via TOPSPIN 4.0.6 software (Bruker Biospin GmbH). Metabolite identification and quantification were performed with AMIX Viewer 3.9.15 (Bruker Biospin GmbH).

Spectra were referenced by setting the TSP signal to 0.0 ppm. Metabolites were identified by comparison with reference spectra from an in-house compound library. For quantification, signal integrals of metabolites were related to the ERETIC signal obtained through external calibration using the PULCON-based ERETIC method [40]. Prior to analysis, background signals were minimised using the AMIX background correction tool. Final metabolite concentrations were normalised to the average dry matter content of the respective intestinal segment and expressed as mmol/kg dry matter (DM).

#### 2.4.3. GC-MS—Gas Chromatography–Mass Spectrometry Analysis

For GC–MS analysis, 100 µL of each sample was supplemented with an internal standard mixture consisting of stable isotope-labelled reference compounds [41]. Samples were subsequently frozen at −80 °C and freeze-dried. Chemical derivatisation of the dried extracts was performed according to previously established procedures [42].

Measurements were conducted using an Agilent 7890B gas chromatograph equipped with an autosampler, an injector (G4513A), and a 5977B mass selective detector (version B.08.00, Agilent, Santa Clara, CA, USA). Metabolite detection was carried out in selected ion monitoring (SIM) mode. The injection volume was 0.5 µL with a split ratio of 1:25. The oven temperature programme started at 70 °C (held for 2 min), followed by an increase of 10 °C/min to 150 °C and then 20 °C/min to a final temperature of 325 °C, which was maintained for 7 min. Data acquisition began after a solvent delay of 5.8 min [43]. All additional GC parameters corresponded to previously published conditions [42].

Quantitative analysis was performed using MassHunter Quantitative Analysis software (version B.08.00, Agilent). Peak areas were normalised against the respective internal standards. Absolute concentrations were determined using external calibration curves ranging from 0.1 to 500 nmol per sample, while metabolites outside this range were evaluated semi-quantitatively. All results were normalised to the amount of extracted sample material. This analytical approach enabled the detection of amino acids and carbohydrates.

### 2.5. Microbiota Profiling Using Next-Generation Sequencing of the 16S rRNA Gene (V3/V4 Region)

Intestinal samples were homogenised with zirconia beads (BioSpec Products) using a MagNALyzer (Roche Diagnostics, Vienna, Austria). Genomic DNA was subsequently isolated with the QIAamp DNA Stool Mini Kit following the manufacturer’s protocol (Qiagen GmbH, Hilden, Germany). DNA concentrations were measured spectrophotometrically, and extracts were stored at −20 °C until further use.

Amplification of the V3/V4 hypervariable region of the bacterial 16S rRNA gene as well as subsequent purification and MiSeq-based sequencing were performed as previously described [44]. The resulting FASTQ files were processed using the Qiime software package (Qiime 1, 2010). Quality filtering was applied using a threshold score of 19, and MID sequences were required to match perfectly. Reverse reads were truncated to 250 bp prior to merging with forward reads.

Chimeric sequences were identified using the slayer algorithm and removed from the dataset. Taxonomic classification of the remaining sequences against entries in the Silva database was conducted with RDP SeqMatch at a 97% operational taxonomic unit (OTU) similarity threshold. Qiime software was used also for calculation of the Chao1, Shannon and Simpson ∝ diversity indices.

### 2.6. Data Analysis and Statistics

Statistical analyses were conducted using SAS Enterprise Guide (version 7.1; SAS Institute Inc., Cary, NC, USA). Residuals of all dependent variables were calculated and assessed for normal distribution (Shapiro–Wilk test), complemented by visual inspection. As the data did not meet the assumptions of normality, non-parametric methods were applied. Group comparisons were therefore performed using the Wilcoxon rank-sum test, with statistical significance set at *p* < 0.05.

## 3. Results

### 3.1. Colonisation of C. jejuni and C. coli in Jejunum and Caecum of Piglets

Both gut segments were co-colonised with a higher amount of *C. coli* in the caecum compared to the jejunum. The number of *C. jejuni* (cfu) was comparable between the gut segments (Table 2). No *Campylobacter* was isolated from the non-inoculated animals.

### 3.2. Metabolome

#### 3.2.1. Detection and Quantification of SCFAs in the Intestinal Digesta

In contrast to the content of the caecum, SCFAs were not detected in the jejunum. As expected, acetate, butyrate and propionate were the most frequently detected SCFAs in the digesta of the caecum. The amount of SCFAs was comparable among the control group (Group 0) and the group with the infected pigs (Group 1) (Table 3).

#### 3.2.2. Detection and Quantification of Amino Acids and Other Metabolites in the Intestinal Digesta

All detected metabolites are listed in Tables S2 and S3. Only four of them were differently abundant in the ileum or caecum of control and experimental pigs. Less cysteine (*p* = 0.0096) and urea (*p* = 0.0312) but more glycine (*p* = 0.0151) were detected in the jejunum of the infected pigs (Group 1) than in pigs of the control group (Group 0). In contrast, there was more 3-hydroxy-butyrate in the caecum of Group 1 animals (*p* = 0.0363) compared to the pigs of Group 0 (Table 4).

### 3.3. Microbiome

#### 3.3.1. Composition and Alpha Diversity of the Microbiota

**Figure 1:**
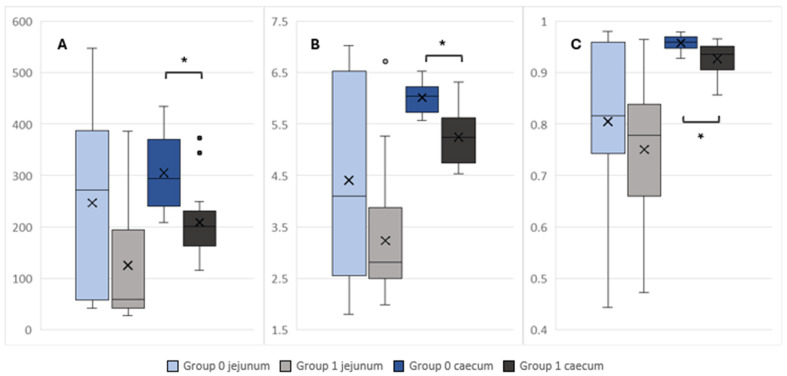
Alpha diversity in samples collected from the jejunal and caecal content compared between groups 0 and 1 (Group 0, control: *n* = 16, Group 1, *C. coli* + *C. jejuni*: *n* = 16). Box plots showing alpha diversity in samples using the Chao1 index (**A**), the Shannon index (**B**) and the Simpson index (**C**). Mean values are marked with an X; statistically significant values are marked with * (*p* < 0.05).

Digesta samples of the caecum in Group 0 showed higher Chao1 (*p* = 0.0007), Shannon (*p* = 0.0005) and Simpson (*p* = 0.0013) alpha diversity indices than in Group 1 (Figure 1). In contrast, alpha diversity was equivalent within the jejunum of infected (Group 1) and control pigs (Group 0).

#### 3.3.2. Bacterial Communities in the Jejunum and Caecum at the Phylum Level

**Figure 2:**
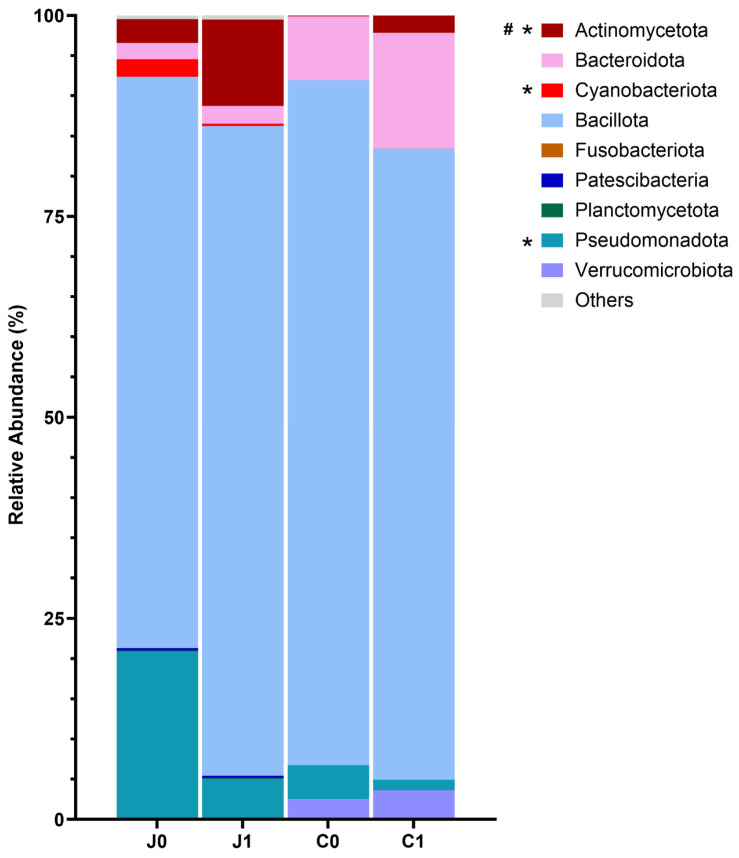
Percent relative abundance of the most abundant phyla in the jejunum and caecum of the infected pigs (Group 1, *n* = 16) compared with the control pigs (Group 0, *n* = 16) (J0 = Group 0 jejunum; J1 = Group 1 jejunum; C0 = Group 0 caecum; C1 = Group 1 caecum). * = statistically significant difference between jejunal values, # = statistically significant difference between caecal values. Standard deviations and *p* values are given in Table S4.

Only phyla with an abundance higher than 1% were considered. In both investigated groups, jejunal and caecal microbiomes were dominated by the phylum Bacillota. In the jejunal digesta, abundance of Bacillota was 71.0% in Group 0 and 80.8% in Group 1. This was followed in descending order by Pseudomonadota (Group 0: 20.7%, Group 1: 4.9%), Actinomycetota (Group 0: 2.9%, Group 1: 10.7%), Bacteroidetes (Bacteroidota) (Group 0: 2.0%, Group 1: 2.2%) and Cyanobacteriota (Group 0: 2.2%, Group 1: 0.3%) (Figure 2, Table S5). In Group 1, a significantly higher prevalence of Actinomycetota (*p* = 0.0282) and Cyanobacteriota (*p* = 0.0315) was observed. Conversely, the relative abundance of Pseudomonadota was notably lower in this group compared to Group 1 (*p* = 0.0042). While the proportion of Bacillota in the infected group exceeded that of the control group in the jejunum, the ratio was the opposite in the caecum (Group 0: 85.2%, Group 1: 78.5%). Bacteriodota was the second most frequently detected phylum (Group 0: 7.9%, Group 1: 14.38%) in the caecum followed by Pseudomonadota (Group 0: 4.2%, Group 1:1.3%) and Actinomycetota (Group 0: 0.1%, Group 1:2.2%) (Figure 2, Table S4). The relative frequency of Actinomycetota in the caecum was, as also in the jejunum, statistically significantly higher in Group 1 than in the control group (*p* = 0.0004), while no further statistical differences were found regarding the phyla.

#### 3.3.3. Bacterial Communities in the Jejunum and Caecum at Class Level

**Figure 3:**
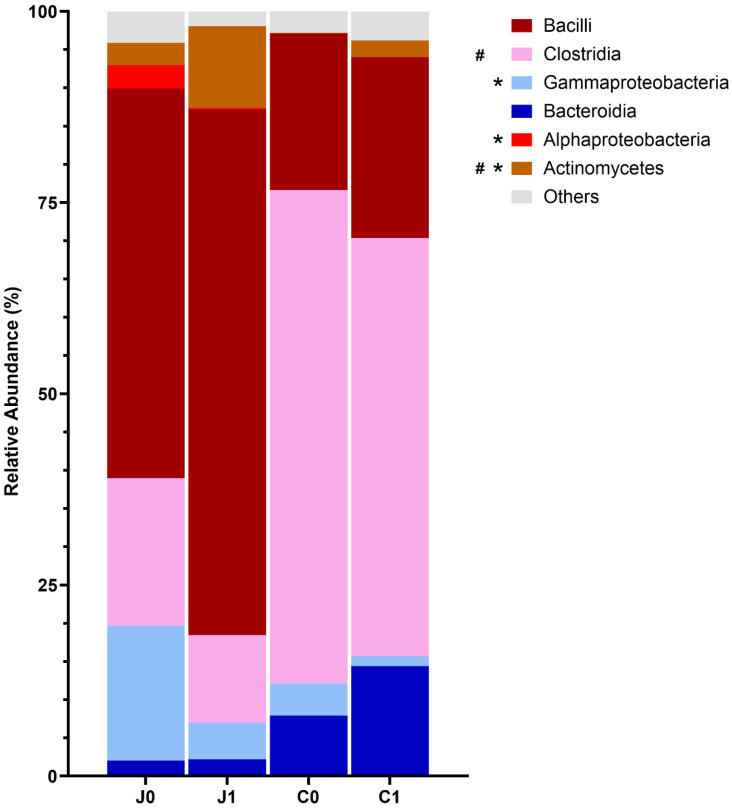
Microbiota composition at class level in the digesta of the jejunum and caecum of Group 0 and Group 1 (Group 0, control: *n* = 16, Group 1, *C. coli* + *C. jejuni*: *n* = 16; J0 = Group 0 jejunum; J1 = Group 1 jejunum; C0 = Group 0 caecum; C1 = Group 1 caecum). Only classes with a relative frequency of more than 1% were taken into account. * = statistically significant difference between jejunal values, # = statistically significant difference between caecal values. Standard deviations and *p* values are given in Table S5.

Out of the six major identified classes of the jejunum Bacilli and Clostridia, both members of the phylum Bacillota predominated in both examined groups (Bacilli: Group 0: 50.9%, Group 1: 68.8%; Clostridia: Group 0: 19.3%, Group 1: 11.4%) without any statistically significant difference. The proportion of Alpha- (Group 0: 3.0%, Group 1: 0.1%, *p* = 0.0021) and Gammaproteobacteria (Group 0: 17.7%, Group 1: 4.8%, *p* = 0.0096) as well as Actinobacteria (Group 0: 2.9%, Group 1: 10.7%, *p* = 0.0312) differed significantly between the studied groups in accordance with their phyla. Bacteroidia (phylum Bacteroidota) was represented in both groups in the jejunum, with a proportion of about 2% (Group 0: 2.0%, Group 1: 2.2%, Figure 3).

The most abundant class in the caecum was the taxon Clostridia in both examined groups (Group 0: 69.1%, Group 1: 54.6%), whereby statistically significantly fewer Clostridia were detected in Group 1 (*p* = 0.0208). Bacilli was the second most common class in the caecum (Group 0: 14.8%, Group 1: 23.7%). Only the class of Actinomycetes differed statistically significantly between the study groups (Group 0: 0.1%, Group 1: 2.2%, *p* < 0.001). As in the jejunum, the proportion in Group 1 was predominant.

Presented here were only taxa with an abundance above 1%. Standard deviations and *p* values are given in Table S5.

#### 3.3.4. Bacterial Communities in the Jejunum and Caecum at Family Level

For the analysis of dissimilarities in jejunal and caecal microbiomes of the two examined groups at family level, only the nine most common families of the digesta were considered. The remaining families are summarised under “others”.

**Figure 4:**
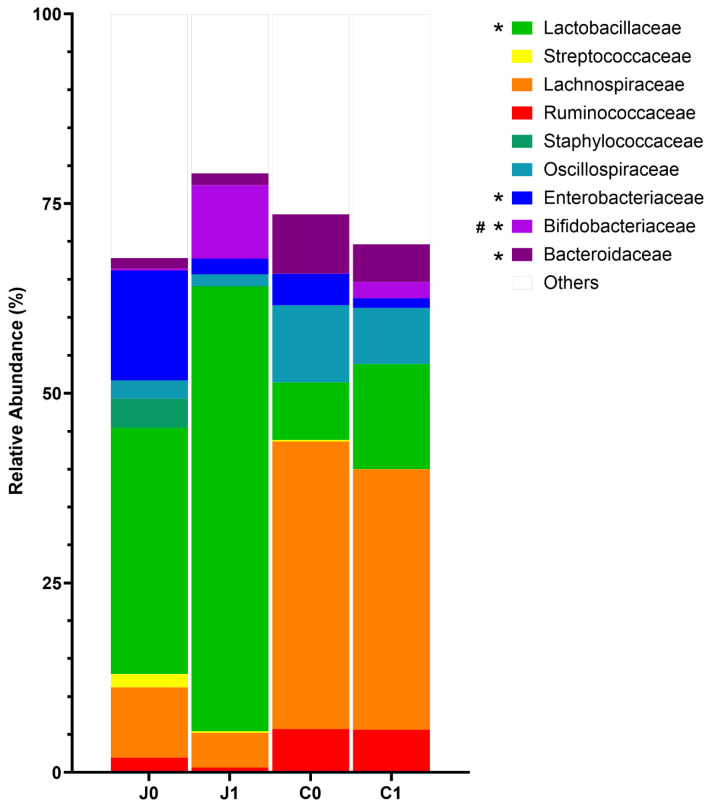
Percent relative abundance of families in the jejunal and caecal digesta of Group 0 and Group 1 (Group 0, control: *n* = 16, Group 1, *C. coli* + *C. jejuni*: *n* = 16; J0 = Group 0 jejunum; J1 = Group 1 jejunum; C0 = Group 0 caecum; C1 = Group 1 caecum). Only the top nine families were taken into account. * = statistically significant difference between jejunal values, # = statistically significant difference between caecal values. Standard deviations and *p* values are given in Table S6.

The most abundant family in the jejunum was Lactobacillaceae (Group 0: 32.4%, Group 1: 58.6%), with significantly higher relative abundances in Group 1 compared to Group 0 (*p* = 0.0076). Bacteroidaceae (Group 0: 1.4%, Group 1: 1.5%, *p* = 0.0306) and Bifidobacteriaceae (Group 0: 0.2%, Group 1: 9.7%, *p* = 0.0050) also showed significantly higher relative abundances in Group 1. In contrast, Enterobacteriaceae were more abundant in Group 0 (Group 0: 14.5%, Group 1: 2.1%, *p* = 0.0025, Figure 4).

In the caecum, Lachnospiraceae had the highest proportion (Group 0: 37.9%, Group 1: 34.4%), followed by Lactobacillaceae (Group 0: 13.3%, Group 1: 13.8%) and Oscillospiraceae (Group 0: 10.2%, Group 1: 7.4%). The only statistically significant difference between Group 0 and Group 1 was noted for Bifidobacteriaceae, which was predominant in the caecal content of Group 1 (Group 0: 1. 0.0%, Group 1: 2.1%, *p* = <0.0001).

## 4. Discussion

*Campylobacter* belongs to the phylum Pseudomonadota, which was created from the reclassification of the proteobacterial class Epsilonproteobacteria and order Campylobacterales. Further changes in the nomenclature of prokaryotes have been made in recent years by the International Committee on Systematics of Prokaryotes (ICPS) [45], so that the names in the literature mentioned below are used in their current version, with the original name in brackets.

*Campylobacter* is considered the most important public health burden worldwide, with poultry as one of the main reservoirs of this zoonotic pathogen. In humans, infection with thermophilic *Campylobacter* manifests with watery to bloody diarrhoea. While colonisation can often be proven in different animal species, the outcome of the infection differs depending on the species infected, the immune status and the infection strain. Colonisation of the pig intestine is generally considered as commensalism, although colostrum-deprived and gnotobiotic piglets develop enteritis after inoculation with *C. jejuni* [16,18,19].

Since, as expected, our pigs did not show any disease symptoms or pathology after inoculation with the two thermophilic *Campylobacter* strains *C. jejuni* ST 1222 and *C. coli* ST 5777, we focussed our study on the comparison of the metabolome and the microbiome of the jejunum and the caecum of infected and uninfected pigs, taking into account the frequency of detection of both strains in the two intestinal sections. On the one hand, it was expected that the intestinal microbiome and the metabolome influence the colonisation with *C. jejuni* and *C. coli* and, on the other hand, changes in the metabolome and microbiome composition due to *Campylobacter* colonisation can be detected in the different segments of the gut.

Given the well-documented similarities between porcine and human intestinal microbiotas [30,46], pigs provide a biologically relevant model for studying *Campylobacter* colonisation. While infection in swine remains asymptomatic, their gastrointestinal physiology and natural susceptibility to *Campylobacter* make them suitable for investigating microbiome and metabolome alterations that can offer insights into mechanisms relevant to human infection.

The colonisation site of *C. jejuni* ST122 and *C. coli* ST5777 in the pig intestine after mono-infection has already been investigated in previous studies. There were no differences between the strains, with both showing higher cfu/mL in ileal and caecal samples than in jejunal samples [39]. Looking at the colonisation in the intestine of co-infected pigs, the abundance of *C. jejuni* in jejunal samples was almost twice as high as that of *C. coli*, although not statistically significant. In the caecal ingesta, *C. coli* was detected at higher relative abundances than *C. jejuni*, although the difference was not very pronounced.

Differences in the distribution patterns may be due to different micro-environmental requirements of the two strains, particularly in the availability of nutrients. In a previous in vitro investigation, the nutrient requirements of both strains were already examined more closely [39]. Of the examined substrates, the *C. coli* strain metabolised acetate, propionate, serine, asparagine, and fucose, while *C. jejuni* only utilised serine for its growth. In the pigs, no difference was detected in the metabolome of the intestinal sections with regard to these substances. In the jejunal content of the infected pigs (Group 1), we found statistically significantly more glycine and less cysteine and urea, while the concentration of 3-hydroxybutyrate increased in the caecum in this group compared to the control group (Group 0).

Glycine is important for glutathione synthesis by the small intestinal mucosa and is a potent cytoprotectant [47]. Little is known about the glycine metabolism of *Campylobacter*. Some *C. jejuni* strains incorporate glycine into the core of lipooligosaccharides (LOS), mimicking ganglosides of the host, which could possibly be important for triggering the Guillain–Barré syndrome [48]. To what extent glycine has a promoting effect on the colonisation of *Campylobacter* ssp., and here especially *C. jejuni*, in the intestine of pigs needs further investigation.

Cysteine is an antioxidant which improves the immunological and the barrier function of the gut and plays a role in rebuilding the gut structure after damage [49]. *C. jejuni* utilises cysteine as a source of sulphur (S) as most *Campylobacter* are unable to assimilate sulphate as the S source [50,51,52]. Consequently, cysteine improves growth of *C. jejuni* [53] but can also be utilised by *C. coli* [54]. The extent to which the consumption of the infectious strains has an influence on the content of cysteine in the jejunum remains speculative, especially since the relative frequency of these strains within the jejunal microbiome is likely to be very low (less than 0.06%), as studies of mono-infections have shown [39].

Auxotrophic behaviour for cysteine is widely distributed within the genus *Bifidobacterium* [55]. Lactobacilli also benefit from supplementation of N-acetyl cysteine, as shown in the increased counts in the gut of weaned pigs associated with an oxidative stress response [56]. We observed statistically significantly increased relative abundance of Lactobacillaceae and Bifidobacteriaceae in the jejunum of the infected pigs compared to the control animals, which might explain the lower content of cysteine in the jejunal digesta because of their utilisation as a growth substrate.

Numerous strains of Bifidobacterium and Lactobacillus are recognized as probiotics [57]. They contribute to health by modulating microbial communities in the gut, reinforcing the integrity of the intestinal barrier, and mitigating oxidative stress [58]. However, despite the increase in the abundance of Bifidobacteriaceae and Lactobacillaceae, colonisation with *Campylobacter* could not be prevented.

Urea is used as a nitrogen source of the gut microbiota for amino acid production in the small intestine [58,59], while it plays only a minor role for growth of most colonic bacteria [60]. Since *C. jejuni* and *C. coli* are not able to hydrolyse urea [61], the lower content in the jejunum of the infected animals cannot be directly linked to the *Campylobacter* colonisation.

The ketone body 3-hydroxybutyrate was found in higher concentrations in the lumen of the caecum of the *Campylobacter* co-infected pigs than in the control group. Colonocytes utilise butyrate to produce acetoacetate and 3-hydroxybutyrate [62]. For the microbiota, 3-hydroxybutyrate is an important source of carbon and energy [63]. Poly-3-hydroxybutyrate is a polymer, which is generated by dehydration and polymerisation of 3-hydroxybutyrate, which forms intracellular granules, being utilised by *C. hepaticus* as a carbon source [64]. However, this metabolic pathway is not available for *C. coli* and *C. jejuni* [65]. Therefore, it is assumed that the 3-hydroxybutyrate concentration in the digesta is not directly influenced by the colonising strains, but it activates butyrate-producing bacteria in the gut lumen [63]. Butyrate is one of the main metabolites in the large intestine and is an important energy source for colonocytes. It maintains gut barrier integrity and positively influences villus height and mucosal epithelial proliferation [32]. Lachnospiraceae belong to the specialists for degradation of plant material, building SFAs. Additionally, members of the family Lactobacillaceae also have butyrogenic capability [66]. Therefore, an influence of the *Campylobacter* infection associated with the increased 3-hydroxybutyrate content in the caecum on the abundance of Lachnospiraceae and Lactobacillaceae would have been expected in this section of the intestine rather than in the jejunum, where no SCFAs were detected.

In contrast to the utilisation of 3-hydroxybutyrate, in vitro and in vivo experiments revealed that Bifidobacteria, reflecting the phylum Actinomycetota [Actinobacteria], were inhibited in growth by 3-hydroxybutyrate [67]. However, despite the higher content of 3-hydoxybutyrate in the caecum of the infected animals, a significantly higher abundance of Bifidobacteriaceae was also detected in Group 1 compared to Group 0.

Since both *Campylobacter* infection strains appear to preferentially colonise the caecum based on the detection frequency, the greatest influence on the microbiome was expected in this section of the intestine.

In fact, alpha diversity indices (Shannon, Chao1, and Simpson) estimating species richness and evenness were lower in caeca of infected animals (Group 1) than in the control group (Group 0), while no differences in the microbial diversity of the jejunum between the experimental groups was observed.

In poultry, inoculation experiments with *Campylobacter* yielded different results of microbial diversity. Awad, Mann [36] as well as Pang, Looft [68] described an increase in the caeca after an infection with *Campylobacter* spp. in chickens, while Rzeznitzeck, Breves [13] observed a lower alpha diversity in infected turkeys. Human patients with post-infectious irritable bowel syndrome after *C. jejuni* enteritis also showed significantly lower diversity in their faeces. The authors suspected a faster transit of the intestinal content in connection with the inflammatory reaction as the cause of the reduction [69]. Yet, all pigs included in our analyses did not show any signs of enteric or systemic disease.

The diversity of gut microbiota varies in dependence on multiple factors like stressors, e.g., environmental temperature, weaning, stocking density, diet, age of the host and breed [70]. To ensure comparability between the investigation groups, 32 cross breed piglets of the same age, obtained by Caesarean section from four sows of the same origin and breed, were raised on similar diets in isolation units under strict hygiene standards and under the same housing conditions.

The higher content of 3-hydroxybutyrate in the caecum of the inoculated pigs has already been mentioned. Newell and Bomhof [71] showed that a ketogenic dietary intervention leads to a decline in the alpha diversity of the intestinal microbiota within a murine model of autism spectrum disorder. Nevertheless, to the authors’ knowledge, there are currently no studies on the influence of 3-hydroxybutyrtate on the microbiome of pigs.

The decrease in alpha diversity in the caecum of the *Campylobacter*-infected pigs might also be linked to the observed increase in the phylum Actinomycetota [Actinobacteria] represented especially by the family Bifidobacteriacea. *Bifidobacterium* ssp. are considered beneficial due to their ability to exclude enteropathogens in the intestine encompassing probiotic properties [72]. In our study, despite the increase in Bifidobacteriaceae in the jejunum and in the caecum of the inoculated pigs (Group 1), colonisation with both *Campylobacter* strains could not be prevented.

At the phylum level, in addition to the higher proportion of Actinomycetota [Actinobacteria], an increase in Cyanobacteriota [Cyanobacteria] was observed in the jejunum of the *Campylobacter*-inoculated pigs (Group 1). Until the discovery of non-photosynthetic Cyanobacteriota [Cyanobacteria] (Melainabacteria), the detection of the phylum in the anterior gut was attributed to the uptake of ingested chloroplasts [73,74]. The relevance of the class of melainabacteria on the colonisation of *Campylobacter* is still questionable. In humans, a positive correlation between gut Cyanobacteriota [Cyanobacteria] abundance and gastrointestinal disease implies its significance [75]. To the authors’ knowledge, no studies on the effects of colonisation with Melainabacteria have been carried out in pigs. However, the higher abundance of Cyanobacteriota [Cyanobacteria] in the jejunum of the *Campylobacter*-inoculated animals did not appear to have any effect on clinical health.

The higher proportion of Actinomycetota [Actinobacteria] and Cyanobacteriota [Cyanobacteria] in the jejunum of the infected pigs seemed to be correlated with a significantly lower abundance of Pseudomonadota. In line with this difference at phylum level, we were also able to demonstrate a statistically lower frequency of the associated classes of Alphaproteobacteria and Gammaproteobacteria and the family of Enterobacteriaceae as representative of the Gammaproteobacteria.

Generally, the phylum Pseudomonadota responds sensitively to environmental factors and is regarded as the most unstable over time among the four main phyla (Bacillota [Firmicutes], Bacteroidetes, Pseudomonadota [Proteobacteria] and Actinomycetota [Actinobacteria]) in the gut microbiota [76]. An increase is regarded as a sign for dysbacteriosis in humans [76] but also in pigs, specifically the increase in the family Enterobacteriaceae, which includes numeric pathogenic bacteria [77].

However, in line with the opinion of Hooks and O’Malley [78], it is doubtful to refer to eubiosis in our *Campylobacter*-infected pigs by reverse implication due to the reduction in Pseudomonadota [Proteobacteria]. The relationship between the colonisation with *Campylobacter* and the decline in Proteobacteriaceae remains unclear, but the simultaneous increase in probiotic members of Lactobacillacea and Bifidobacteriaceae can be speculated as the cause of the decline. In addition, we observed a higher abundance of Bacteroidaceae in the jejunum of pigs infected with *Campylobacter* (Group 1). As a member of the class Bacteroidia they are adapted to a low oxygen environment and increase when oxygen-tolerant Pseudomonadota [Proteobacteria] including Enterobacteriaceae decrease. Due to their property to produce SCFAs, mainly acetic and propionic acid, growth of Enterobacteriacea is directly reduced by lowering the pH in the gut [79].

## 5. Conclusions

We determined the classification levels from phylum to family to understand the interaction of *C. jejuni* and *C. coli* on the porcine gut microbiome using 16S amplicon sequencing. At the same time, the changes in metabolome composition in the jejunum and caecum should be linked to the microbiome composition.

In conclusion, there were only a few variations in the metabolite composition in the intestinal sections as a result of the *Campylobacter* infection. Of 44 substances detected, only 3 in the jejunum and 1 in the caecum differed statistically significantly between the test groups. However, the connection between the change in the metabolite composition in the respective intestinal sections and the colonisation with *Campylobacter* raise new questions. Nonetheless, the findings of this investigation indicate, despite the absence of clinical symptoms, there were changes in the intestinal microbiota as a result of the *Campylobacter* infection, leading to a decrease in diversity in the caeca. Surprisingly, those bacterial families seemed to benefit from the infection, known for their probiotic properties, some of which are thought to be effective against *Campylobacter* infections. The results of our study indicate that neither Bifidobacteriaceae nor Lactobacillaceae displace the inoculated *Campylobacter* strains from the intestine.

Regarding the design of the study, there are a few points of criticism: firstly, we did not differentiate between mucosa-associated and luminal-associated microbiota of the two intestinal segments. According to Zhang et al. [79], the luminal microbiota primarily metabolises and digests nutrients, while the mucosal microbiome is more involved in the immune function. Consequently, there are differences in the spatial distribution of the microbiota. According to Kelly, Daly et al. [25], the large intestine’s mucosa is specifically colonised by microaerophilic *Campylobacter* spp., whereas the luminal space is primarily inhabited by obligate anaerobes such as Veillonellaceae, Ruminococcaceae, Lachnospiraceae, and Prevotellaceae.

Additionally, we determined the classification levels from phylum to family and attempted to correlate them to the results of the metabolome determination. But Wakita, Shimomura [80] demonstrated that lower taxonomic ranks are more strongly associated with the metabolome.

However, the present findings highlight the need for further studies to clarify the interaction between *Campylobacter* colonisation and the presence of Enterobacteriaceae in pigs. Enterobacteriaceae are of major relevance in pig production, as they include important intestinal pathogens and are already subject to targeted control measures, such as farm-level interventions against *E. coli* and the EU-wide *Salmonella* control programme. Understanding whether and how these interventions may influence *Campylobacter* colonisation could contribute to the development of more integrated control strategies. In this context, the results of the present study provide a valuable basis for future research addressing the complex interactions within the intestinal microbiota of pigs.

## Figures and Tables

**Table 1:** Infection scheme of the two animal groups (Group 0, control: *n* = 16, Group 1, *C. coli* + *C. jejuni*: *n* = 16).

Group	Number of Piglets	Inoculation	Inoculation Dose
0	16	10 mL nutrient broth	0
1	16	10 mL nutrient broth with *C. coli* +10 mL nutrient broth with *C. jejuni*	1.04 × 10^10^ *C. coli* 1.3 × 10^9^ *C. jejuni*

**Table 2:** Quantification of the administered *Campylobacter* strains (cfu/mL) in the jejunum and caecum at necropsy during week 12 of life, four weeks post-inoculation.

Intestinal Section	*Campylobacter* Counts (cfu/mL)	
	*C. coli* ST 5777	*C. jejuni* ST122
Jejunum	5.34 × 10^2^ *	1.35 × 10^4^ *
Caecum	4.04 × 10^5^	9.27 × 10^4^

* Statistically significant difference between the marked values (*p* < 0.05). Average concentrations within the intestinal material of Group 1 (*n* = 16): *C. coli* ST 5777 + *C. jejuni* ST 122.

**Table 3:** Concentration of SCFAs in the digesta in the caecum.

Short Chain Fatty Acids (SCFA)	Group 0 (mmol/kg Digesta)	Group 1 (mmol/kg Digesta)
Acetate	38.42	38.92
Butyrate	8.35	8.16
Caproate	0.39	0.40
Formate	0.18	0.12
Isovalerate	0.20	0.25
Propionate	3.13	3.73
Valerate	0.43	0.55

Average concentrations within the intestinal material are reported in mmol/kg (Group 0, control: *n* = 16, Group 1, *C. coli* + *C. jejuni*: *n* = 16). Standard deviations are given in Table S3.

**Table 4:** Concentration of cysteine, glycine, 3-hydroxybutyrate and urea in the digesta of jejunum and caecum.

Metabolites (mmol/kg Digesta)	Jejunum		Group 1 (mmol/kg Digesta)	
	Group 0	Group 1	Group 0	Group 1
Cysteine	0.9 *	0.04 *	0.03	0.03
Glycine	4.87 *	8.58 *	0.24	0.22
3-hydroxybutyrate	0.0005	0.0004	0.0002 *	0.006 *
Urea	0.23 *	0.10 *	0.003	0.003

* Statistically significant difference between the marked values (*p* < 0.05). Average concentrations within the intestinal material are reported in mmol/kg (Group 0, control: *n* = 16, Group 1, *C. coli* + *C. jejuni*: *n* = 16). Standard deviations are given in Tables S1 and S2.

## Data Availability

The original data presented in the study are openly available in NCBI Short Read Archive under accession number PRJNA1453471.

## Supplementary Materials

The following supporting information can be downloaded at: https://www.mdpi.com/article/10.3390/microorganisms14050945/s1, Table S1: Concentration of amino acids in the jejunal and caecal digesta.; Table S2: Concentration of metabolites in the jejunal and caecal digesta.; Table S3: Concentration of short-chain fatty acids in caecal digesta.; Table S4: Percent relative abundance of the most abundant phyla in the jejunum and caecum of the infected pigs (Group 1, *n* = 16) compared with the control pigs (Group 0, *n* = 16) with standard deviations and *p* values.; Table S5: Percent relative abundance of the most abundant classes in the jejunum and caecum of the infected pigs (Group 1, *n* = 16) compared with the control pigs (Group 0, *n* = 16) with standard deviations and *p* values.; Table S6: Percent relative abundance of the most abundant families in the jejunum and caecum of the infected pigs (Group 1, *n* = 16) compared with the control pigs (Group 0, *n* = 16) with standard deviations and *p* values.

## References

[1] European Food Safety Authority (EFSA), European Centre for Disease Prevention and Control (ECDC) (2024). The European union one health 2023 zoonoses report. EFSA J..

[2] Robert Koch-Institut (2021). Infektionsepidemiologisches Jahrbuch Meldepflichtiger Krankheiten für 2020. https://edoc.rki.de/handle/176904/8766.2.

[3] Harvey R.B., Young C.R., Ziprin R.L., Hume M.E., Genovese K.J., Anderson R.C., Droleskey R.E., Stanker L.H., Nisbet D.J. (1999). Prevalence of *Campylobacter* spp. isolated from the intestinal tract of pigs raised in an integrated swine production system. J. Am. Vet. Med. Assoc..

[4] von Altrock A., Louis A.L., Rosler U., Alter T., Beyerbach M., Kreienbrocks L., Waldmann K.-H. (2006). The bacteriological and serological prevalence of *Campylobacter* spp. and *Yersinia enterocolitica* in fattening pig herds in Lower Saxony. Berl. Munch. Tierarztl. Wochenschr..

[5] von Altrock A., Hamedy A., Merle R., Waldmann K.H. (2013). *Campylobacter* spp.—Prevalence on pig livers and antimicrobial susceptibility. Prev. Vet. Med..

[6] Young C.R., Harvey R., Anderson R., Nisbet D., Stanker L.H. (2000). Enteric colonisation following natural exposure to *Campylobacter* in pigs. Res. Vet. Sci..

[7] Dearlove B.L., Cody A.J., Pascoe B., Meric G., Wilson D.J., Sheppard S.K. (2016). Rapid host switching in generalist *Campylobacter* strains erodes the signal for tracing human infections. ISME J..

[8] van Vliet A.H., Ketley J.M. (2001). Pathogenesis of enteric *Campylobacter* infection. J. Appl. Microbiol..

[9] Awad W.A., Aschenbach J.R., Ghareeb K., Khayal B., Hess C., Hess M. (2014). *Campylobacter jejuni* influences the expression of nutrient transporter genes in the intestine of chickens. Vet. Microbiol..

[10] Awad W.A., Molnár A., Aschenbach J.R., Ghareeb K., Khayal B., Hess C., Liebhart D., Dublecz K., Hess M. (2015). *Campylobacterinfection* in chickens modulates the intestinal epithelial barrier function. Innate Immun..

[11] Awad W.A., Smorodchenko A., Hess C., Aschenbach J.R., Molnar A., Dublecz K., Khayal B., Pohl E.E., Hess M. (2015). Increased intracellular calcium level and impaired nutrient absorption are important pathogenicity traits in the chicken intestinal epithelium during *Campylobacter jejuni* colonization. Appl. Microbiol. Biotechnol..

[12] Humphrey S., Chaloner G., Kemmett K., Davidson N., Williams N., Kipar A., Humphrey T., Wigley P. (2014). *Campylobacter jejuni* is not merely a commensal in commercial broiler chickens and affects bird welfare. mBio.

[13] Rzeznitzeck J., Breves G., Rychlik I., Hoerr F.J., von Altrock A., Rath A., Rautenschlein S. (2022). The effect of *Campylobacter jejuni* and *Campylobacter coli* colonization on the gut morphology, functional integrity, and microbiota composition of female turkeys. Gut Pathog..

[14] Rath A., Rautenschlein S., Rzeznitzeck J., Breves G., Hewicker-Trautwein M., Waldmann K.H., von Altrock A. (2021). Impact of *Campylobacter* spp. on the Integrity of the Porcine Gut. Animals.

[15] Bratz K., Bucker R., Golz G., Zakrzewski S.S., Janczyk P., Nockler K., Alter T. (2013). Experimental infection of weaned piglets with *Campylobacter coli*—Excretion and translocation in a pig colonisation trial. Vet. Microbiol..

[16] Babakhani F.K., Bradley G.A., Joens L.A. (1993). Newborn piglet model for campylobacteriosis. Infect. Immun..

[17] Boosinger T.R., Powe T.A. (1988). *Campylobacter jejuni* infections in gnotobiotic pigs. Am. J. Vet. Res..

[18] de Vries S.P., Linn A., Macleod K., MacCallum A., Hardy S.P., Douce G., Watson E., Dagleish M.P., Thompson H., Stevenson A. (2017). Analysis of *Campylobacter jejuni* infection in the gnotobiotic piglet and genome-wide identification of bacterial factors required for infection. Sci. Rep..

[19] Vítovec J., Koudela B., Štěrba J., Tomancová I., Matyáš Z., Vladík P. (1989). The gnotobiotic piglet as a model for the pathogenesis of *Campylobacter jejuni* infection. Zentralblatt Bakteriol..

[20] Alemka A., Corcionivoschi N., Bourke B. (2012). Defense and adaptation: The complex inter-relationship between *Campylobacter jejuni* and mucus. Front. Cell. Infect. Microbiol..

[21] Dasti J.I., Tareen A.M., Lugert R., Zautner A.E., Gross U. (2010). *Campylobacter jejuni*: A brief overview on pathogenicity-associated factors and disease-mediating mechanisms. Int. J. Med. Microbiol..

[22] Hofreuter D., Novik V., Galan J.E. (2008). Metabolic diversity in *Campylobacter jejuni* enhances specific tissue colonization. Cell Host Microbe.

[23] Bescucci D.M., Moote P.E., Ortega Polo R., Uwiera R.R.E., Inglis G.D. (2020). *Salmonella enterica* Serovar Typhimurium Temporally Modulates the Enteric Microbiota and Host Responses To Overcome Colonization Resistance in Swine. Appl. Environ. Microbiol..

[24] Gresse R., Chaucheyras Durand F., Duniere L., Blanquet-Diot S., Forano E. (2019). Microbiota Composition and Functional Profiling Throughout the Gastrointestinal Tract of Commercial Weaning Piglets. Microorganisms.

[25] Kelly J., Daly K., Moran A.W., Ryan S., Bravo D., Shirazi-Beechey S.P. (2017). Composition and diversity of mucosa-associated microbiota along the entire length of the pig gastrointestinal tract; dietary influences. Environ. Microbiol..

[26] Panah F.M., Lauridsen C., Hojberg O., Jensen H.E., Nielsen T.S. (2023). Composition of mucus- and digesta-associated bacteria in growing pigs with and without diarrhea differed according to the presence of colonic inflammation. BMC Microbiol..

[27] Burnham P.M., Hendrixson D.R. (2018). *Campylobacter jejuni*: Collective components promoting a successful enteric lifestyle. Nat. Rev. Microbiol..

[28] Adhikari B., Kim S.W., Kwon Y.M. (2019). Characterization of Microbiota Associated with Digesta and Mucosa in Different Regions of Gastrointestinal Tract of Nursery Pigs. Int. J. Mol. Sci..

[29] Holman D.B., Brunelle B.W., Trachsel J., Allen H.K. (2017). Meta-analysis To Define a Core Microbiota in the Swine Gut. mSystems.

[30] Wang H., Xu R., Zhang H., Su Y., Zhu W. (2020). Swine gut microbiota and its interaction with host nutrient metabolism. Anim. Nutr..

[31] Zoetendal E.G., Raes J., van den Bogert B., Arumugam M., Booijink C.C.G.M., Troost F.J., Bork P., Wels M., De Vos W.M., Kleerebezem M. (2012). The human small intestinal microbiota is driven by rapid uptake and conversion of simple carbohydrates. ISME J..

[32] Silva Y.P., Bernardi A., Frozza R.L. (2020). The Role of Short-Chain Fatty Acids From Gut Microbiota in Gut-Brain Communication. Front. Endocrinol..

[33] Kim H.B., Borewicz K., White B.A., Singer R.S., Sreevatsan S., Tu Z.J., Isaacson R.E. (2011). Longitudinal investigation of the age-related bacterial diversity in the feces of commercial pigs. Vet. Microbiol..

[34] Mach N., Berri M., Estelle J., Levenez F., Lemonnier G., Denis C., Leplat J., Chevaleyre C., Billon Y., Doré J. (2015). Early-life establishment of the swine gut microbiome and impact on host phenotypes. Environ. Microbiol. Rep..

[35] Awad W.A., Dublecz F., Hess C., Dublecz K., Khayal B., Aschenbach J.R., Hess M. (2016). *Campylobacter jejuni* colonization promotes the translocation of *Escherichia coli* to extra-intestinal organs and disturbs the short-chain fatty acids profiles in the chicken gut. Poult. Sci..

[36] Awad W.A., Mann E., Dzieciol M., Hess C., Schmitz-Esser S., Wagner M., Hess M. (2016). Age-Related Differences in the Luminal and Mucosa-Associated Gut Microbiome of Broiler Chickens and Shifts Associated with *Campylobacter jejuni* Infection. Front. Cell. Infect. Microbiol..

[37] Thibodeau A., Fravalo P., Yergeau E., Arsenault J., Lahaye L., Letellier A. (2015). Chicken Caecal Microbiome Modifications Induced by *Campylobacter jejuni* Colonization and by a Non-Antibiotic Feed Additive. PLoS ONE.

[38] Yan W., Zhou Q., Yuan Z., Fu L., Wen C., Yang N., Sun C. (2021). Impact of the gut microecology on *Campylobacter* presence revealed by comparisons of the gut microbiota from chickens raised on litter or in individual cages. BMC Microbiol..

[39] Rath A., Rautenschlein S., Rzeznitzeck J., Lalk M., Methling K., Rychlik I., Peh E., Kittler S., Waldmann K.-H., von Altrock A. (2022). Investigation on the colonisation of *Campylobacter* strains in the pig intestine depending on available metabolites. Comp. Immunol. Microbiol. Infect. Dis..

[40] Wider G., Dreier L. (2006). Measuring protein concentrations by NMR spectroscopy. J. Am. Chem. Soc..

[41] Leonard A., Mohlis K., Schluter R., Taylor E., Lalk M., Methling K. (2020). Exploring metabolic adaptation of *Streptococcus pneumoniae* to antibiotics. J. Antibiot..

[42] Dorries K., Schlueter R., Lalk M. (2014). Impact of antibiotics with various target sites on the metabolome of *Staphylococcus aureus*. Antimicrob. Agents Chemother..

[43] Surabhi S., Jachmann L.H., Lalk M., Hammerschmidt S., Methling K., Siemens N. (2021). Bronchial epithelial cells accumulate citrate intracellularly in response to pneumococcal hydrogen peroxide. ACS Infect. Dis..

[44] Kubasova T., Davidova-Gerzova L., Merlot E., Medvecky M., Polansky O., Gardan-Salmon D., Quesnel H., Rychlik I. (2017). Housing Systems Influence Gut Microbiota Composition of Sows but Not of Their Piglets. PLoS ONE.

[45] Oren A. (2024). On validly published names, correct names, and changes in the nomenclature of phyla and genera of prokaryotes: A guide for the perplexed. npj Biofilms Microbiomes.

[46] Negretti N.M., Ye Y., Malavasi L.M., Pokharel S.M., Huynh S., Noh S., Klima C.L., Gourley C.R., A Ragle C., Bose S. (2020). A porcine ligated loop model reveals new insight into the host immune response against *Campylobacter jejuni*. Gut Microbes.

[47] Yang Z., Liao S.F. (2019). Physiological Effects of Dietary Amino Acids on Gut Health and Functions of Swine. Front. Vet. Sci..

[48] Dzieciatkowska M., Brochu D., van Belkum A., Heikema A.P., Yuki N., Houliston R.S., Richards J.C., Gilbert M., Li J. (2007). Mass spectrometric analysis of intact lipooligosaccharide: Direct evidence for O-acetylated sialic acids and discovery of O-linked glycine expressed by *Campylobacter jejuni*. Biochemistry.

[49] Liao S.F. (2021). Invited Review: Maintain or Improve Piglet Gut Health around Weanling: The Fundamental Effects of Dietary Amino Acids. Animals.

[50] Garvis S.G., Tipton S.L., Konkel M.E. (1997). Identification of a functional homolog of the *Escherichia coli* and *Salmonella typhimurium cysM* gene encoding O-acetylserine sulfhydrylase B in *Campylobacter jejuni*. Gene.

[51] Hitchcock N., Kelly D.J., Hitchcock A., Taylor A.J. (2022). Cysteine Biosynthesis in *Campylobacter jejuni*: Substrate Specificity of CysM and the Dualism of Sulfide. Biomolecules.

[52] Vorwerk H., Mohr J., Huber C., Wensel O., Schmidt-Hohagen K., Gripp E., Josenhans C., Schomburg D., Eisenreich W., Hofreuter D. (2014). Utilization of host-derived cysteine-containing peptides overcomes the restricted sulphur metabolism of *Campylobacter jejuni*. Mol. Microbiol..

[53] Tejera N., Crossman L., Pearson B., Stoakes E., Nasher F., Djeghout B., Poolman M., Wain J., Singh D. (2020). Genome-Scale Metabolic Model Driven Design of a Defined Medium for *Campylobacter jejuni* M1cam. Front. Microbiol..

[54] Mohammed K.A., Miles R.J., Halablab M.A. (2004). The pattern and kinetics of substrate metabolism of *Campylobacter jejuni* and *Campylobacter coli*. Lett. Appl. Microbiol..

[55] Ferrario C., Duranti S., Milani C., Mancabelli L., Lugli G.A., Turroni F., Mangifesta M., Viappiani A., Ossiprandi M.C., van Sinderen D. (2015). Exploring Amino Acid Auxotrophy in *Bifidobacterium bifidum* PRL2010. Front. Microbiol..

[56] Xu C.C., Yang S.F., Zhu L.H., Cai X., Sheng Y.S., Zhu S.W., Xu J.X. (2014). Regulation of N-acetyl cysteine on gut redox status and major microbiota in weaned piglets. J. Anim. Sci..

[57] Hill C., Guarner F., Reid G., Gibson G.R., Merenstein D.J., Pot B., Morelli L., Canani R.B., Flint H.J., Salminen S. (2014). Expert consensus document. The International Scientific Association for Probiotics and Prebiotics consensus statement on the scope and appropriate use of the term probiotic. Nat. Rev. Gastroenterol. Hepatol..

[58] Wang Y., Kirpich I., Liu Y., Ma Z., Barve S., McClain C.J., Feng W. (2011). *Lactobacillus rhamnosus* GG treatment potentiates intestinal hypoxia-inducible factor, promotes intestinal integrity and ameliorates alcohol-induced liver injury. Am. J. Pathol..

[59] Krone J.E.C., Agyekum A.K., Ter Borgh M., Hamonic K., Penner G.B., Columbus D.A. (2019). Characterization of urea transport mechanisms in the intestinal tract of growing pigs. Am. J. Physiol. Gastrointest. Liver Physiol..

[60] Fuller M., Reeds P. (1998). Endogenous nitrogen in the gut. Annu. Rev. Nutr..

[61] Matsuda M., Moore J.E. (2004). Urease-positive thermophilic *Campylobacter* species. Appl. Environ. Microbiol..

[62] Darcy-Vrillon B., Cherbuy C., Morel M.T., Durand M., Duee P.H. (1996). Short chain fatty acid and glucose metabolism in isolated pig colonocytes: Modulation by NH_4_^+^. Mol. Cell. Biochem..

[63] Satoh T., Sasaki K. (2024). 3-Hydroxybutyrate could serve as a principal energy substrate for human microbiota. Med. Hypotheses.

[64] Muller-Santos M., Koskimaki J.J., Alves L.P.S., de Souza E.M., Jendrossek D., Pirttila A.M. (2021). The protective role of PHB and its degradation products against stress situations in bacteria. FEMS Microbiol. Rev..

[65] Van T.T.H., Lacey J.A., Vezina B., Phung C., Anwar A., Scott P.C., Moore R.J. (2019). Survival Mechanisms of *Campylobacter hepaticus* Identified by Genomic Analysis and Comparative Transcriptomic Analysis of In Vivo and In Vitro Derived Bacteria. Front. Microbiol..

[66] Fu X., Liu Z., Zhu C., Mou H., Kong Q. (2019). Nondigestible carbohydrates, butyrate, and butyrate-producing bacteria. Crit. Rev. Food Sci. Nutr..

[67] Ang Q.Y., Alexander M., Newman J.C., Tian Y., Cai J., Upadhyay V., Turnbaugh J.A., Verdin E., Hall K.D., Leibel R.L. (2020). Ketogenic Diets Alter the Gut Microbiome Resulting in Decreased Intestinal Th17 Cells. Cell.

[68] Pang J., Looft T., Zhang Q., Sahin O. (2023). Deciphering the Association Between *Campylobacter* Colonization and Microbiota Composition in the Intestine of Commercial Broilers. Microorganisms.

[69] Jalanka J., Gunn D., Singh G., Krishnasamy S., Lingaya M., Crispie F., Finnegan L., Cotter P., James L., Nowak A. (2023). Postinfective bowel dysfunction following *Campylobacter enteritis* is characterised by reduced microbiota diversity and impaired microbiota recovery. Gut.

[70] Patil Y., Gooneratne R., Ju X.H. (2020). Interactions between host and gut microbiota in domestic pigs: A review. Gut Microbes.

[71] Newell C., Bomhof M.R., Reimer R.A., Hittel D.S., Rho J.M., Shearer J. (2016). Ketogenic diet modifies the gut microbiota in a murine model of autism spectrum disorder. Mol. Autism.

[72] Gueimonde M., Margolles A., de los Reyes-Gavilan C.G., Salminen S. (2007). Competitive exclusion of enteropathogens from human intestinal mucus by Bifidobacterium strains with acquired resistance to bile—A preliminary study. Int. J. Food Microbiol..

[73] Hofer U. (2013). Bacterial evolution: Getting to the bottom of Cyanobacteria. Nat. Rev. Microbiol..

[74] Di Rienzi S.C., Sharon I., Wrighton K.C., Koren O., Hug L.A., Thomas B.C., Goodrich J.K., Bell J., Spector T.D., Banfield J.T. (2013). The human gut and groundwater harbor non-photosynthetic bacteria belonging to a new candidate phylum sibling to Cyanobacteria. eLife.

[75] Hu C., Rzymski P. (2022). Non-Photosynthetic Melainabacteria (Cyanobacteria) in Human Gut: Characteristics and Association with Health. Life.

[76] Shin N.R., Whon T.W., Bae J.W. (2015). Proteobacteria: Microbial signature of dysbiosis in gut microbiota. Trends Biotechnol..

[77] Sun J., Du L., Li X., Zhong H., Ding Y., Liu Z., Ge L. (2019). Identification of the core bacteria in rectums of diarrheic and non-diarrheic piglets. Sci. Rep..

[78] Hooks K.B., O’Malley M.A. (2017). Dysbiosis and Its Discontents. mBio.

[79] Zhang L., Wu W., Lee Y.K., Xie J., Zhang H. (2018). Spatial Heterogeneity and Co-occurrence of Mucosal and Luminal Microbiome across Swine Intestinal Tract. Front. Microbiol..

[80] Wakita Y., Shimomura Y., Kitada Y., Yamamoto H., Ohashi Y., Matsumoto M. (2018). Taxonomic classification for microbiome analysis, which correlates well with the metabolite milieu of the gut. BMC Microbiol..

