# Supplementary material for: Consequences of Campylobacter jejuni and Campylobacter coli Colonisation of Piglets on Gut Microbiota and Microbial Metabolites
Source: Microorganisms. 2026 Apr 22;14(5):945. doi: 10.3390/microorganisms14050945 (PMC13209335; doi:10.3390/microorganisms14050945)
Supplement: Supplementary file 1 [file microorganisms-14-00945-s001.zip › microorganisms-4234337-supplementary.pdf]

Table S1. Concentration of amino acids in the jejunal and caecal digesta. The values are given as mean values in mmol/kg intestinal content with standard deviation and p values (Group 0, control: n = 16. Group 1. *C. coli* + *C. jejuni*: n = 16).

Amino acids	Jejunum		P values	Caecum		P values
	Group 0	Group 1		Group 0	Group 1	
4-hydroxyproline	0.01 ± 0.00	0.01 ± 0.00	0.2436	0.01 ± 0.00	0.01 ± 0.01	0.2206
5-oxoproline	0.21 ± 0.13	0.14 ± 0.04	0.2280	0.13 ± 0.11	0.10 ± 0.05	0.8358
Alanine	1.14 ± 0.63	0.68 ± 0.20	0.0786	0.59 ± 0.85	0.35 ± 0.15	0.7487
Asparagine	0.45 ± 0.31	0.24 ± 0.08	0.1591	0.06 ± 0.04	0.08 ± 0.05	0.3558
Aspartate	0.47 ± 0.39	0.18 ± 0.07	0.0930	0.68 ± 0.44	0.78 ± 0.51	0.5847
Fumarate	0.03 ± 0.02	0.02 ± 0.01	0.0552	0.02 ± 0.01	0.02 ± 0.01	0.8951
Glutamate	0.99 ± 0.72	0.48 ± 0.18	0.2280	1.74 ± 0.86	1.77 ± 0.79	0.8065
Glutamine	0.78 ± 0.68	0.39 ± 0.14	0.1383	0.20 ± 0.10	0.14 ± 0.07	0.0797
Isoleucine	0.46 ± 0.37	0.24 ± 0.11	0.2280	0.11 ± 0.08	0.08 ± 0.05	0.6109
Leucine	0.32 ± 0.21	0.23 ± 0.09	0.5401	0.07 ± 0.05	0.06 ± 0.04	0.6109
Lysine	0.92 ± 0.67	0.51 ± 0.20	0.1854	0.34 ± 0.20	0.34 ± 0.22	0.9519
Malate	0.04 ± 0.04	0.02 ± 0.01	0.1184	0.07 ± 0.06	0.05 ± 0.02	0.7487
Methionine	0.23 ± 0.19	0.13 ± 0.05	0.4177	0.07 ± 0.04	0.05 ± 0.03	0.3365
Ornithine	0.04 ± 0.08	0.04 ± 0.03	0.0855	0.11 ± 0.07	0.17 ± 0.14	0.3365
Phenylalanine	0.44 ± 0.30	0.28 ± 0.10	0.3954	0.07 ± 0.04	0.06 ± 0.04	0.4856
Proline	0.60 ± 0.43	0.37 ± 0.15	0.6212	0.13 ± 0.09	0.09 ± 0.04	0.1809
Pyruvate	0.04 ± 0.06	0.02 ± 0.01	0.7971	0.02 ± 0.01	0.02 ± 0.02	0.7203
Serine	0.63 ± 0.49	0.33 ± 0.14	0.1383	0.14 ± 0.09	0.12 ± 0.06	0.5847
Threonine	0.47 ± 0.38	0.18 ± 0.08	0.0661	0.15 ± 0.09	0.15 ± 0.08	0.8065
Thyrosine	0.32 ± 0.21	0.21 ± 0.07	0.3738	0.09 ± 0.05	0.07 ± 0.05	0.3558
Tryptophane	0.09 ± 0.06	0.06 ± 0.02	0.2599	0.02 ± 0.01	0.01 ± 0.01	0.0935
Valine	0.60 ± 0.52	0.28 ± 0.12	0.0935	0.14 ± 0.09	0.12 ± 0.07	0.7203
Cysteine	0.09 ± 0.05	0.05 ± 0.02	0.0096	0.03 ± 0.03	0.03 ± 0.02	0.4397
Glycine	4.87 ± 3.12	8.59 ± 3.62	0.0151	0.25 ± 0.14	0.22 ± 0.10	0.4624

Table S2. Concentration of metabolites in the jejunal and caecal digesta. The values are given as mean values in mmol/kg intestinal content with standard deviation and p values (Group 0, control: n = 16. Group 1. C. coli + C. jejuni: n = 16).

Others	Jejunum		P values	Caecum		P values
	Group 0	Group 1		Group 0	Group 1	
2-oxoglutarate	0	0	0.1697	0	0	0.2134
Beta-alanine	0.03 ± 0.03	0.02 ± 0.01	0.3954	0.08 ± 0.05	0.03 ± 0.05	0.0899
Citrate	0.05 ± 0.07	0.01 ± 0.01	0.5799	0.02 ± 0.02	0.02 ± 0.01	0.7630
Fructose	0.26 ± 0.46	0.01 ± 0.01	0.0721	0.39 ± 1.31	0.05 ± 0.04	0.2067
Glucose	10.65 ± 11.66	18.27 ± 13.35	0.0930	0.62 ± 0.47	0.39 ± 0.19	0.2828
Histidine	0.26 ± 0.22	0.11 ± 0.04	0.1605	0.05 ± 0.03	0.05 ± 0.03	0.9249
Lactate	19.68 ± 12.72	24.69 ± 11.66	0.2131	5.16 ± 12.13	2.23 ± 1.38	0.6923
Maltose	1.10 ± 2.34	0.31 ± 0.73	0.2272	0	0	1.0
Myo-Inositole	0.20 ± 0.14	0.29 ± 0.30	0.5665	0.02 ± 0.02	0.02 ± 0.03	0.2826
Succinate	0.14 ± 0.10	0.12 ± 0.06	0.8588	10.74 ± 12.31	9.60 ± 9.34	0.8358
Sucrose	0.07 ± 0.14	0.00 ± 0.00	0.1278	0.02 ± 0.05	0.01 ± 0.00	0.0522

3-hydroxybutyrate	0.00 ± 0.00	0.00 ± 0.00	0.6068	0.00 ± 0.00	0.01 ± 0.01	0.0363
Urea	0.23 ± 0.16	0.10 ± 0.08	0.0312	0.00 ± 0.00	0.00 ± 0.00	0.3080

Table S3. Concentration of short-chain fatty acids in caecal digesta. The values are given as mean values in mmol/kg intestinal content with standard deviation and p values (Group 0, control: n = 16. Group 1, C. coli + C. jejuni: n = 16).

Short-chain fatty acids (SCFAs)	Group 0 (mmol/kg digesta)	Group 1 (mmol/kg digesta)	P values
Acetate	38.42 ± 7.59	38.92 ± 7.07	0.8358
Butyrate	8.35 ± 2.88	8.16 ± 2.91	0.8653
Caproate	0.39 ± 0.38	0.40 ± 0.44	0.6109
Formate	0.18 ± 0.25	0.12 ± 0.03	0.4178
Isovalerate	0.20 ± 0.08	0.25 ± 0.15	0.4856
Propionate	3.13 ± 1.08	3.73 ± 1.30	0.1809
Valerate	0.43 ± 0.14	0.55 ± 0.43	0.8951

Table S4. Percent relative abundance of the most abundant phyla in the jejunum and caecum of the infected pigs (Group 1, n = 16) compared with the control pigs (Group 0, n = 16) with standard deviations and p values.

Phylum	Relative abundance in the jejunum (mean values + standard deviation)		P values	Relative abundance in the caecum (mean values + standard deviation)		P values
	Group 0 (Abundance in %)	Group 1 (Abundance in %)		Group 0 (Abundance in %)	Group 1 (Abundance in %)	
Bacillota	71.05 ± 23.72	80.81 ± 13.30	0.5143	85.21 ± 10.46	78.47 ± 19.69	0.7073
Pseudomonadota	20.71 ± 21.79	4.95 ± 8.25	0.0042	4.20 ± 7.91	1.35 ± 2.26	0.1605
Actinomycetota	2.97 ± 3.51	10.74 ± 12.02	0.0282	0.10 ± 0.06	2.17 ± 2.61	0.0004
Bacteroidota	2.03 ± 1.66	2.21 ± 3.81	0.1208	7.93 ± 9.08	14.38 ± 16.47	0.1267
Cyanobacteriota	2.17 ± 4.31	0.30 ± 0.60	0.0315	0.00 ± 0.00	0.01 ± 0.05	0.5897
Verrucomicrobiota	0.16 ± 0.20	0.09 ± 0.07	1.0	2.56 ± 4.35	3.61 ± 4.46	0.3107
Fusobacteriota	0.01 ± 0.05	0	0.1503	0	0	1.0
Patescibacteria	0.33 ± 0.54	0.28 ± 0.47	0.3527	0.0003 ± 0.0007	0.001 ± 0.004	0.5786
Planctomycetota	0.12 ± 0.31	0.12 ± 0.30	0.8601	0.0002 ± 0.0007	0	0.3329

Table S5. Percent relative abundance of the most abundant classes in the jejunum and caecum of the infected pigs (Group 1, n = 16) compared with the control pigs (Group 0, n = 16) with standard deviations and p values.

Class	Relative abundance in the jejunum (mean values + standard deviation)		P values	Relative abundance in the caecum (mean values + standard deviation)		P values
	Group 0 (Abundance in %)	Group 1 (Abundance in %)		Group 0 (Abundance in %)	Group 1 (Abundance in %)	
Bacilli	50.95 ± 23.72	68.79 ± 13.30	0.2131	14.80 ± 10.46	23.69 ± 19.69	0.1605
Clostridia	19.30 ± 21.79	11.44 ± 8.25	0.4177	69.14 ± 7.91	54.63 ± 2.26	0.0208
Negativicutes	0.79 ± 0.83	0.56 ± 1.26	0.0455	0.03 ± 0.03	0.01 ± 0.01	0.0488
Alphaproteobacteria	3.05 ± 3.51	0.15 ± 12.02	0.0021	0.0001 ± 0.06	0.0005 ± 2.61	0.5377
Epsilonproteobacteria	0.00 ± 0.00	0.22 ± 0.51	0.0377	0	0	1.0
Gammaproteobacteria	17.66 ± 1.66	4.80 ± 3.81	0.0096	4.21 ± 9.08	1.35 ± 16.47	0.1605
Deltaproteobacteria	0.10 ± 0.18	0.01 ± 0.03	0.0216	0.004 ± 0.01	0	0.0148
Actinomycetes	2.92 ± 4.31	10.71 ± 0.60	0.0312	0.07 ± 0.004	2.16 ± 0.05	0.001
Thermoleophilia	0.02 ± 0.05	0.01 ± 0.02	0.3744	0.0002 ± 0.0007	0	0.3329
Bacteroidia	2.02 ± 0.05	2.21 ±	0.1280	7.93	14.38	0.1267

Table S6. Percent relative abundance of the most abundant families in the jejunum and caecum of the infected pigs (Group 1, n = 16) compared with the control pigs (Group 0, n = 16) with standard deviations and p values.

Family	Relative abundance in the jejunum (mean values + standard deviation)		P values	Relative abundance in the caecum (mean values + standard deviation)		P values
	Group 0 jejunum (Abundance in %)	Group 1 jejunum (Abundance in %)		Group 0 caecum (Abundance in %)	Group 1 caecum (Abundance in %)	
Lactobacillaceae	32.45 ± 26.91	58.59 ± 18.90	0.0076	13.26 ± 4.34	13.82 ± 8.90	0.0504
Streptococcaceae	1.77 ± 3.67	0.25 ± 0.30	0.1267	0.21 ± 0.76	0.02 ± 0.02	0.7457
Staphylococcaceae	3.83 ± 13.10	0.14 ± 0.26	0.4233	0.01 ± 0.03	0.003 ± 0.01	0.3964
Lachnospiraceae	9.27 ± 9.54	4.59 ± 6.33	0.1605	37.89 ± 13.04	34.36 ± 11.62	0.3135
Oscillospiraceae	2.44 ± 2.67	1.49 ± 2.60	0.1375	10.16 ± 4.54	7.40 ± 3.77	0.0721
Ruminococcaceae	1.96 ± 2.24	0.63 ± 1.10	0.0632	5.74 ± 5.25	5.64 ± 2.82	0.2770
Enterobacteriaceae	14.48 ± 20.59	2.05 ± 5.80	0.0025	4.17 ± 7.92	1.28 ± 2.19	0.1383
Bifidobacteriaceae	0.22 ± 0.37	9.70 ± 12.45	0.0050	0.02 ± 0.04	2.14 ± 2.62	0.0001
Bacteriodaceae	1.42 ± 1.20	1.54 ± 3.27	0.0306	7.82 ± 8.98	4.97 ± 8.32	0.2661
